# Two Vanilloid Ligand Bindings Per Channel Are Required to Transduce Capsaicin-Activating Stimuli

**DOI:** 10.3389/fnmol.2019.00302

**Published:** 2020-01-09

**Authors:** Ting-Yi Liu, Ying Chu, Hao-Ruei Mei, Dennis Chang, Huai-Hu Chuang

**Affiliations:** Institute of Molecular Biology, Academia Sinica, Taipei, Taiwan

**Keywords:** TRPV1, capsaicin, anandamide, 2-APB activation, stoichiometry-dependent non-linear activation

## Abstract

The tetrameric capsaicin receptor transient receptor potential vanilloid 1 (TRPV1) in mammals has evolved the capability to integrate pain signal arising from harmful temperature and chemical irritants. The four repetitions of TRPV1 subunits result in an ion channel with excellent pain sensitivity, allowing this ionotropic receptor to differentiate graded injuries. We manipulated the stoichiometry and relative steric coordination of capsaicin-bound structures at the molecular level to determine the rules by which the receptor codes pain across a broad range of intensities. By introducing capsaicin-insensitive S512F mutant subunits into the TRPV1 channel, we found that binding of the first ligand results in low but clear channel activation. Maximal agonist-induced activation is already apparent in tetramers harboring two or three wild-type TRPV1 subunits, which display comparable activity to wild-type tetramer. The non-vanilloid agonist 2-aminoethoxydiphenyl borate (2-APB) differs from that of capsaicin in the TRPV1 channel opening mechanism activating all S512F-mutated TRPV1 channels. Two or more wild-type TRPV1 subunits are also required for full anandamide-induced channel activation, a cannabinoid that shares overlapping binding-pocket to capsaicin. Our results demonstrate that the stoichiometry of TRPV1 activation is conserved for two types of agonists.

## Introduction

Transient receptor potential vanilloid 1 (TRPV1) is a cation-permeating channel assembled from four identical subunits that surround the ion-conducting pore. Each subunit contains six transmembrane α-helices (S1–S6) that span the lipid bilayer (Clapham, 2003; Liao et al., 2013). TRPV1 responds to physical challenges like harmful temperatures and chemicals (protons, irritants, and inflammatory mediators), converting them into electrical activity in sensory neurons for chemical or thermal nociception (Tominaga et al., 1998; Caterina et al., 2000; Davis et al., 2000; Macpherson et al., 2005; Siemens et al., 2006; Nieto-Posadas et al., 2011; Marrone et al., 2017). Capsaicin is the best known vanilloid ligand for TRPV1, a chemical in chili peppers that can induce cation influx and primary sensory neuronal action potentials, typically resulting in a burning sensation in mammals (Wood et al., 1988; Caterina et al., 1997).

Different stimulatory factors bind to distinct sites on TRPV1. Mutational and molecular modeling studies have shown that capsaicin can interact with residues Y511, S512, T550, and E570 on the intracellular side of rat TRPV1, resulting in channel opening (Yang and Zheng, 2017). Extracellular protons and spider toxin interact with the outer pore domain of TRPV1 for channel activation (Jordt et al., 2000; Bae et al., 2016). 2-aminoethoxydiphenyl borate (2-APB) can activate multiple members of the TRP channel family, including TRPV1, but the 2-APB binding site in TRPV1 is distinct from its vanilloid binding pocket (Hu et al., 2004; Singh et al., 2018). Unlike ligand binding sites, the cytosolic N- or C-terminal of rat and human TRPV1 is responsible for modulating the strength of channel responses to strong stimuli such as capsaicin, with oxidation and phosphorylation of calmodulin targeting those regions (Bhave et al., 2003; Rosenbaum et al., 2004; Chuang and Lin, 2009). “Computational” integration within neurons dictates TRPV1 response amplitudes subsequent to signal convergence.

The TRPV1 channel comprises four repeats of the same subunit, thereby facilitating quantification of combinations and intensities of environmental insults. However, how modulation and activation of each subunit lead to channel gating are not known. Most structural and modeling studies have focused on fully stimulated channels and assume consistent activity of all subunits (Hanson et al., 2015; Ohbuchi et al., 2016). Furthermore, there is limited data about the requirement for multiple agonist-bound subunits of TRPV1 to transduce stronger pain signal (Hui et al., 2003; Hazan et al., 2015).

We used a functional approach to determine how many capsaicin-sensitive subunits are required to conduct maximum capsaicin signal in cells. We quantified intracellular Ca^2+^ increases in HEK293T cells by micro-fluorometry, which has frequently been employed in TRPV1 channel studies (Fenwick et al., 2017; Vanden Abeele et al., 2019). We mixed capsaicin-insensitive S512F mutants and wild-type TRPV1 subunits to tune the capacity for ligand-induced channel opening. We found that the ability of combinatory channels to relay capsaicin excitation was dictated by the number of S512F subunits they harbored. While TRPV1 receptor activation depends on compositional stoichiometry, it can be further complicated by heterogeneity of arrangements of mutant and wild-type subunits. We overcame this complication by constructing linked tetrameric receptors in which the number and arrangement of mutant and wild-type subunits within the TRPV1 channels were consistent, vastly simplifying our mechanistic analyses of the activation of differentially arranged TRPV1 tetramers. Our findings reveal that multiple capsaicin-bound subunits are required to drive maximal channel opening.

## Materials and Methods

### Molecular Biology-Multimeric Rat TRPV1 Constructs

We used QuikChange site-directed mutagenesis to generate single-point mutants by overlap extension PCR using Phusion polymerase (New England Biolab). Tetramers were constructed by linking four rat TRPV1 genes with the inter-subunit hepta-peptide linker ANENGDA between the N and C termini, followed by restriction digestion and ligation (Robinson and Sauer, 1998; Chen et al., 2013).

### Cell Culture and Heterologous Expression

Human embryonic kidney 293T (HEK293T) cells were maintained in MEM/EBSS (HyClone) supplemented with 10% fetal bovine serum (FBS, Gibco), 100 U/ml penicillin and 100 μg/ml streptomycin (Lonza). HEK293T cells were transfected with 1–2 μg wild-type, mutant or tetramer receptor plasmids using Avalanche-Omni transfection reagent (EZ Biosystems). After transfection (24–36 h), cells were re-seeded on 96-well plates coated with poly-D-lysine (0.1 mg/ml) and collagen (55 μg/ml), before conducting calcium-imaging assays on the next day. HEK293 cells stably expressing rat TRPV1 were maintained in MEM/EBSS (HyClone) supplemented with 10% fetal bovine serum (FBS, Gibco), 100 U/ml penicillin and 100 μg/ml streptomycin (Lonza), and 0.6 mg/ml active G418 salfate (GoldBio).

### Ratiometric Calcium Imaging

All calcium imaging experiments were conducted at 22°C. Cells were loaded with the Ca^2+^ indicators Fura-2-AM, Fura-4F-AM or MagFura-2 (2 μM, Thermo Fisher Scientific) in 1.7-fold OR-2 solution (8.5 mM HEPES, 140.3 mM NaCl, 3.4 mM KCl, 1.7 mM MgCl_2_, and 1 mM CaCl_2_, pH 7.4) or the calcium replacement buffer (1 mM/10 mM SrCl_2_). For most experiments, we used Fura-2 for recording unless indicated otherwise. All samples were incubated at 30°C for 3 h and then cells were washed in a bath of 1 mM EGTA solution to remove the Ca^2+^ indicator before being transferred to the recording bath. Fluorescence data were acquired by capturing UV excitation wavelength (340 or 380 nm) at a frame rate of 2 s with 20–50 ms exposure time, using an EMCCD camera (Photometrics, Evolve) driven by the Slidebook 6 digital microscopy software (Intelligent Imaging Innovations). Over 200 cells in the recording fields were included for data analysis. The “±” notation in this report represents standard error of the mean (SEM), calculated according to the sample numbers given in the respective figure legends and tables. One-way ANOVA followed by Tukey’s Honestly Significant Difference *post hoc* test for multiple comparisons or Student’s *t*-test was applied to determine statistical significance and is indicated as *P < 0.05, **P < 0.01, or ***P < 0.001. The “a, b, c…” or “A, B, C…” labeling in figures denotes homogenous subsets of results based on multiple comparisons of *post hoc* tests at a mean significance level of 0.05.

### Sodium Dodecyl Sulfate Polyacrylamide Gel Electrophoresis (SDS-PAGE) Analysis and Western Blotting

Following transfection, HEK293T cells were lysed with lysis buffer containing 0.5% Triton X-100, 0.5% NP-40 and protease inhibitor. The cell lysates were mixed with 6× SDS non-reducing sample buffer or 6× SDS reducing sample buffer containing 2% dithiothreitol and 5% 2-mercaptoethanol. The lysates were resolved by 7.5% non-reducing SDS-PAGE before transferring the proteins onto a PVDF Transfer Membrane (Millipore). The membrane was incubated in blocking buffer (5% nonfat milk in Tris-buffered saline with 0.05% Tween 20) containing anti-rat TRPV1 antibody (GeneTex; 1:5,000 dilution) or anti-GAPDH antibody (Santa Cruz Biotechnology, Santa Cruz, CA, USA; 1:5,000 dilution). Proteins were visualized using a secondary anti-rabbit HRP-conjugated antibody (Thermo Fisher Scientific, Waltham, MA, USA; 1:20,000 dilution). PVDF membranes were visualized by supersignal West Femto chemiluminescence substrate (Thermo Fisher Scientific, Waltham, MA, USA), and the blot images were acquired using BioSpectrum 810 (UVP).

### Whole Cell Recordings

Experiments were executed at room temperature (22°C). Transiently-transfected cells were plated onto poly-D-lysine-coated coverslips (0.1 mg/ml) and prepared for electrophysiological analysis. Whole cell recordings were made using 1–3 MΩ fire-polished recording electrodes. The extracellular solution contained 10 mM HEPES, 140 mM NaCl, 1 mM MgCl_2_, and 1 mM CaCl_2_ (pH = 7.4 with NaOH). The intracellular solutions contained 10 mM HEPES, 130 mM Na gluconate, 10 mM NaCl, 1 mM Mg(gluconate)_2_, and 0.1 mM Ethylene glycol-bis(2-aminoethylether)-N,N,N′,N′-tetraacetic acid (EGTA; pH = 7.4 with NaOH). Either 300 μM 2-APB or 100 μM capsaicin was dissolved in extracellular solution for activation. Patchmaster software was used to acquire and analyze data. Cells were stimulated every second from −100 to 80 mV at 180 ms.

### Ionomycin Calibration

Ionomycin (Thermo Fisher Scientific, Waltham, MA, USA; Abcam) was used to calibrate derived calcium concentrations from ratios of emitted fluorescence given by 340/380 nm UV excitation determined with Fura-2AM and Fura-4F. Ten EGTA-buffered standards with different free calcium concentrations were generated by mixing calcium-free buffer (10 mM EGTA in 100 mM KCl, 30 mM MOPS, pH 7.2) and 39 μM calcium buffer (10 mM CaEGTA in 100 mM KCl, 30 mM MOPS, pH 7.2), and then preparing a serial top-down dilution with Ionomycin (2 μM). Stable cell lines pretreated with Fura Ca^2+^ indicator dyes were incubated with each buffer at 30°C before recording according to the method described under ratiometric calcium imaging above.

## Results

### Real-Time Monitoring of Capsaicin-Elicited Influxes of Calcium or Strontium Ions

**Figure 1:**
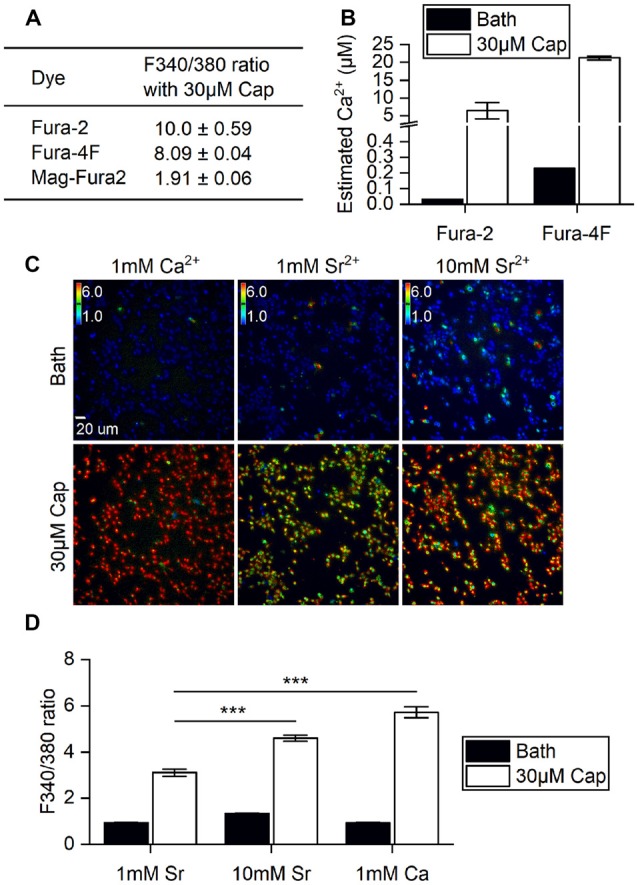
Ratiometric determination of concentrations of divalent cations in different ionic bath solutions. **(A)** F340/380 ratio of bath solutions (Bath) and in response to 30 μM capsaicin (Cap) application, as detected by different dyes (*n* = 3 experiments). **(B)** The ratios of Fura-2 and Fura-4F were converted to intracellular Ca^2+^ concentration increments based on standard curves. Note that the Y axis is broken at 0.5–4 μM Ca^2+^. The basal intracellular Ca^2+^ concentrations indicated by different Fura dyes were (Ca^2+^) = 0.033 μM (Fura-2) and 0.230 ± 0.001 μM (Fura-4F). **(C)** Representative images of stable cell line transient receptor potential vanilloid 1 (TRPV1) responses upon 30 μM capsaicin treatment. Cells were immersed in bath solutions of 1 mM Ca^2+^, 1 mM Sr^2+^ or 10 mM Sr^2+^ with Fura-2. Colored bars indicate ratio changes ranging from 1 to 6. **(D)** Bar graphs show the F340/380 ratio before (Bath) and after 30 μM capsaicin treatment of transiently-transfected HEK293T cells (*n* = 3). ***P < 0.001.

First, we determined if the stimulatory effects of capsaicin on TRPV1 and its ion permeability can be monitored by changes in Fura fluorescence within HEK293T cells. A variety of Fura dyes—Fura-2 (Kd = 145 nM), Fura-4F (Kd = 770 nM) and MagFura-2 (Kd = 25 μM)—together cover the entire physiological range of Ca^2+^ for cross-referencing. Direct application to cells of 30 μM capsaicin evoked saturated cellular ionic influxes through the ligand-gated TRPV1 channel (Yang et al., 2015). TRPV1 activation by 30 μM capsaicin resulted in large-scale Ca^2+^ influx, with a 1.9-fold increase as measured by Mega-Fura2 dye, an 8-fold increase by Fura-4F, and a 10-fold increase by Fura-2 (Figure 1A). However, only Fura-2 and Fura-4F exhibited large-scale fluorescence 340/380 ratio increments, allowing detection of Ca^2+^ influx in the micromolar range. Ratiometric outputs represent estimated intracellular Ca^2+^ concentrations of 21.2 ± 0.5 μM for Fura-4F and 6.5 ± 2.3 μM for Fura-2 upon 30 μM capsaicin treatment (Figure 1B).

TRPV1 can conduct divalent metal ions from the IIA group (Bouron et al., 2015). Both Ca^2+^ and Sr^2+^ ion influxes can be monitored based on concentration-dependent Fura-2 fluorescence (Kwan and Putney, 1990). We substituted Ca^2+^ with Sr^2+^ to explore any differences in cation entry that had not been measured in the higher resolution system. Strontium (Sr^2+^) influxes were dependent on extracellular Sr^2+^ concentration, with 10 mM Sr^2+^ resulting in a significant increase in the F340/F380 ratio over 1 mM Sr^2+^ in bath solution upon capsaicin stimulation (Student’s *t*-test, *p* < 0.001). However, Sr^2+^ influx was markedly lower than that found for 1 mM extracellular Ca^2+^ with 30 μM capsaicin (Figures 1C,D). These data show that increases in capsaicin-induced cations primarily occurred *via* the TRPV1 channel that allows extracellular ions to permeate plasma membranes. Also, the channel exhibits greater ratio increments for Ca^2+^ rather than Sr^2+^ permeability. Together, our experiments show that Fura-2 dye imaging represents an appropriate non-invasive technique for monitoring vanilloid stimulation of TRPV1 responses in live cells, and demonstrates the combined effects of permeability and availability of the permeating cations.

### Replacement of TRPV1 Subunits With the S512F Mutant Specifically Reduces Capsaicin-Evoked Channel Activity

**Figure 2:**
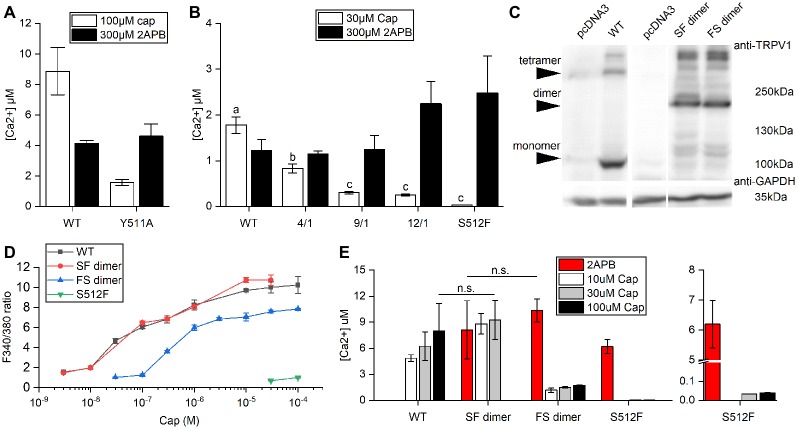
Comparison of Ca^2+^ influxes between wild-type and mutant TRPV1 receptors. **(A)** Response of wild-type TRPV1 or the Y511A mutant to induced activation by 2-aminoethoxydiphenyl borate (2-APB) or capsaicin treatment (Ca^2+^ influxes monitored by Fura-4F). **(B)** Different ratios of the S512F mutant to wild-type TRPV1 plasmids were mixed (4/1, 9/1 and 12/1) for transfection to assess Ca^2+^ influxes. **(C)** Western blot showing protein expressions of the wild-type and dimeric TRPV1 channels. Arrows point to TRPV1 tetrameric aggregates, dimers, and monomers. GAPDH, loading control. **(D)** HEK293T cells expressing wild-type TRPV1, S512F monomer, or SF or FS dimers were treated with increasing doses of capsaicin. Capsaicin-induced activity was similar for SF dimer and wild-type TRPV1. **(E)** The responses of dimeric TRPV1 constructs to capsaicin and 2-APB relative to those of monomeric wild-type and S512F mutant channels. The responses are presented as intracellular Ca^2+^ concentrations (*n* = 3–4 experiments). Note that the Y axis of the right-most graph (S512F) is broken at 0.1–5 μM Ca^2+^. n.s., nonsignificant.

The wild-type TRPV1 channel is formed by four identical subunits. Previously, it was reported that a tandem tetramer comprising one wild-type subunit (Tyr511) and three Y511A mutant subunits exhibited capsaicin-mediated TRPV1 activation (Hazan et al., 2015), indicating that binding of one capsaicin molecule to a TRPV1 tetramer is sufficient to fully open the TRPV1 channel. However, we found that the Y511A mutant still conducted calcium [(Ca^2+^) = 1.58 ± 0.2 μM] under high-concentration capsaicin stimulation, suggesting that the Y511A mutation is not completely capsaicin insensitive (Figure 2A).

Consequently, we focused on S512F mutants for this study. The serine residue at position 512 (Ser512) is located where the first intracellular loop transits into the third transmembrane segment. Ser512 was previously identified as being critical to capsaicin interaction (Jordt and Julius, 2002; Ohkita et al., 2012; Yang et al., 2015). We constructed the capsaicin-insensitive mutant S512F to establish how many capsaicin-binding subunits are required for TRPV1 activation. In contrast to the Y511A mutant, we did not record any capsaicin-induced responses in terms of cytoplasmic Ca^2+^ flux for the S512F mutant [(Ca^2+^) = 0.034 μM; Figure 2B]. Thus, a mutant TRPV1 channel comprised of four non-binding vanilloid-insensitive S512F mutant subunits may act as a silent receptor. To fully probe the functionality of the mutant TRPV1 channels, we tested the structurally unrelated non-vanilloid but full TRPV1 agonist 2-APB in terms of S512F mutant channel activation (Hu et al., 2004; Singh et al., 2018). The S512F mutant channel was activated by 2-APB more strongly than were capsaicin- and 2-APB-activated wild-type TRPV1 channels (Figure 2B). Therefore, the S512F subunit is functional as long as suitable ligands are supplied.

Reintroduction of wild-type subunits into these non-functional S512F tetramers might potentially rescue their capsaicin-mediated activity. We mixed wild-type subunits with increasing ratios of S512F subunits to assemble TRPV1 channels with one wild-type plus three mutant subunits. We found that by increasing the ratio of S512F mutant to wild-type subunits in the mix, capsaicin activation in terms of Ca^2+^ influx was progressively compromised to a low basal level [F/S_(mutant/wildtype)_ = 12/1 (Ca^2+^) = 0.25 ± 0.02 μM; Figure 2B]. No significant difference in Ca^2+^ influx was detected for the F/S ratios of 9/1 and 12/1. Thus, basal Ca^2+^ conductance may be attributable to the TRPV1 channels containing only one wild-type subunit.

Next, we generated dimeric TRPV1 constructs expressing a fusion protein of one wild-type (denoted as “S”) and one S512F (denoted as “F”) subunit (Figure 2C), thus allowing us to assay TRPV1 channels comprising two wild-type and two mutant subunits. Expression of dimeric receptors FS or SF resulted in TRPV1 channel opening by capsaicin, with SF dimers exhibiting ion-conducting efficacy comparable to wild-type TRPV1 (Student’s *t*-test, *p* = 0.762; Figures 2D,E). SF or FS dimers assembled as pores lined by two dimerized dimers (i.e., SFSF or FSFS) exhibited similar maximal levels of Ca^2+^ entry under 2-APB activation (Student’s *t*-test, *p* = 0.572; Figure 2E). Thus, provided that two of the four channel subunits are wild type, the final receptor complex can transduce the full range of agonist signal.

### Tetrameric TRPV1 Requires Two or More Wild-Type Subunits to Fully Transduce Signal

**Figure 3:**
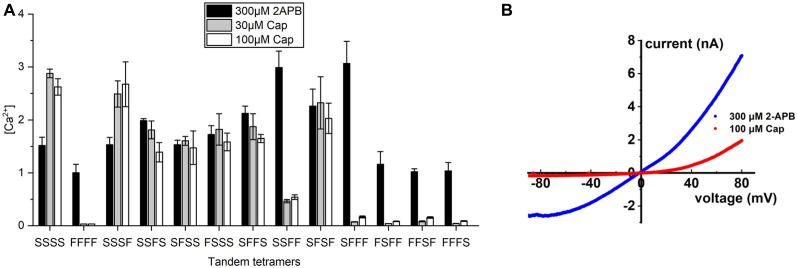
Calcium signal transduction efficiency of tetrameric TRPV1 channels with fixed numbers of S512F subunits. **(A)** Comparison of Ca^2+^ influxes for different tetrameric TRPV1 channels with S512F mutant subunits in various positions of the tetramer in response to treatment with 300 μM 2-APB, or 30 μM or 100 μM capsaicin (*N* = 3 experiments). **(B)** Voltage-current traces show the impact of 100 μM capsaicin (red line) or 300 μM 2-APB (blue line) treatment on HEK293T cells transiently-expressing the SFFF construct.

These results prompted us to generate covalently-linked subunits, allowing us to create stoichiometrically-fixed tandem TRPV1 receptors comprising wild-type and S512F mutant subunits and to compare their activities based on their differing numbers and configurations of capsaicin-sensitive subunits. As controls, we applied 300 μM of the pan-TRPV agonist 2-APB to activate all tetramers and found that receptor activations were strong and uniform across all tandem tetramers irrespective of the identity of the amino acid at position 512, suggesting that covalently-linked tetramers are functional (Figure 3A). We then applied 30 or 100 μM capsaicin to fully stimulate individual tetramers and then measured their activation. The tetramer solely comprising mutant subunits (FFFF) exhibited almost no response to either concentration of capsaicin [(Ca^2+^) = 0.033 μM and Figure 3A], which is consistent with the result from transfection of the S512F transgene (Figure 1B). All mono-S tetramers (SFFF, FSFF, FFSF, and FFFS) presented very low responses to capsaicin stimulation, even at 100 μM capsaicin (Figure 3A), with a maximal average peak response of 0.17 ± 0.02 μM for the SFFF configuration. Even when we quantified cell responses individually, maximal Ca^2+^ concentrations attained in any cell expressing mono-S tetramers never presented more than 0.5 μM free cytosolic calcium. Electrophysiological data also showed that, even at 100 μM capsaicin, the SFFF tetramer did not achieve full activation, unlike for 2-APB treatment (Figure 3B). Interestingly, double-S tetramers (SFFS and SFSF) or triple-S tetramers (SSFS, SFSS, and FSSS) all attained full activation at 30 μM capsaicin, and no further enhancement could be detected by using 100 μM capsaicin. Activations of double- and triple-S tetramers were comparable to those of the wild-type SSSS tetramer, i.e., within the range of 1–3 μM (Ca^2+^). Only the double-S tetramer SSFF presented a lower response of ~0.5 μM (Ca^2+^). However, that outcome is not due to the number of wild-type subunits as the other double-S tetramers (SFFS and SFSF) exhibited high (Ca^2+^) responses. Together, these results suggest that TRPV1 channel activation was far lower than the maximal attainable activity when only one capsaicin-binding site was occupied by vanilloids. However, a pair of occupied capsaicin binding sites was sufficient to elevate channel efficacy to the maximal level displayed by wild-type tetramer.

### Two Ligands of the Partial Agonist AEA Must Also Be Bound to TRPV1 For it to Exert Maximum Channel Efficacy

**Figure 4:**
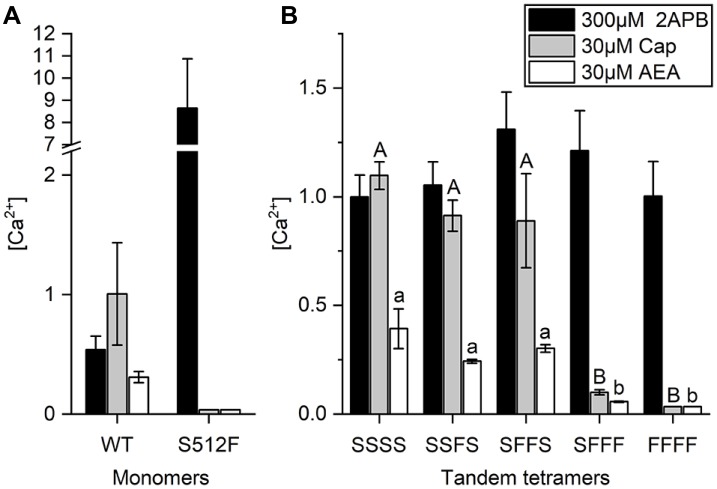
AEA-induced activation requires two wild-type TRPV1 subunits in the TRPV1 channel. **(A)** Comparison of wild-type and S512F mutant TRPV1 responses to 2-APB, AEA, and capsaicin. Note that the Y axis of the left graph is broken at 2.2–7 μM Ca^2+^. **(B)** Activation of tetrameric TRPV1 constructs harboring 0–4 wild-type subunits induced by capsaicin, AEA, or 2-APB (*n* = 3–5 experiments). 2-APB response levels among tandem tetramers are equal based on ANOVA followed by Tukey’s Honestly Significant Difference *post hoc* test for multiple comparisons. The “A, B, C…” and “a, b, c…” labeling in the plot denotes homogenous subsets of tandem tetramers response to capsaicin and AEA, respectively.

Anandamide (AEA), a membrane lipid known to activate cannabinoid receptors, can also activate TRPV1 for pain regulation (Zou and Kumar, 2018). AEA application was previously shown to reduce specific binding of the vanilloid resinferatoxin (RTX) on TRPV1, and Y511A mutation abrogates AEA-induced TRPV1 channel activation, indicating that the AEA binding site is close to the vanilloid binding pocket (Jordt and Julius, 2002). We found that whereas wild-type TRPV1 responded to 30 μM AEA treatment, with an intracellular (Ca^2+^) = 0.31 ± 0.05 μM, introduction of the S512F mutation reduced the AEA-induced TRPV1 response to an almost undetectable level [(Ca^2+^) = 0.036 μM], i.e., equivalent to the level observed for capsaicin stimulation, which supports the importance of residue S512 for AEA activation (Figure 4A).

Next, we determined if AEA stimulation also requires two active binding sites in TRPV1 channels. As a partial agonist of TRPV1, AEA did not elicit as strong a response in wild-type TRPV1 channels as capsaicin (Figure 4B), even when we administered AEA at its maximal dose for activation (Fenwick et al., 2017). Upon stimulation by 30 μM AEA, the calcium concentration in SFFF cells [(Ca^2+^) = 0.06 μM] was considerably less than that in cells expressing either SSSS [(Ca^2+^) = 0.39 ± 0.09 μM], SSFS [(Ca^2+^) = 0.24 ± 0.01 μM], or SFFS [(Ca^2+^) = 0.30 ± 0.02 μM] tetramers (Figure 4B). As a comparative control, we found that 2-APB activated all TRPV1 channels, irrespective of their composition and capsaicin-induced TRPV1 channel activation when two or more wild-type subunits were present. These data support that, like capsaicin, AEA requires two or more wild-type subunits for full activation of the TRPV1 channel.

## Discussion

Pungent vanilloid compounds isolated from natural sources exhibit a wide range of potencies and activities, manifesting in humans as desirable culinary “spiciness” to chemical deterrence. Capsaicin is a lipophilic molecule that binds to the transmembrane domain of TRPV1, presumably aided by local lipid interactions provided by the membrane environment (Cao et al., 2013a,b; Hanson et al., 2015). The capsaicin receptor TRPV1 serves as a critical node in nociception, representing an attractive target for modifying pain sensations (Basso et al., 2019; Lee et al., 2019). Thus, further research into the TRPV1 channel would enhance our understanding of the thresholds or dynamic range of pain sensation and perhaps reveal potential therapeutic applications.

In a previous electrophysiology experiment, binding of one capsaicin molecule to TRPV1 was shown to open the channel, provided that the membrane was held at a sufficiently positive polarization potential (Hazan et al., 2015). In that same study, the mutant TRPV1 Y511A was reported to have lost its capsaicin sensitivity. Besides, Hazan et al. (2015) demonstrated that the Y511A mutant is still activated by heat and/or protons. Due to technical limitations, voltages could only be applied in those experiments within a range tolerated by the membrane. To overcome this difficulty, we sought bath solutions that might augment cation influxes without excessive voltage drive in intact cells. We used non-invasively-loading divalent cation-sensitive indicators to permit time-lapse Ca^2+^ imaging, which allowed us to track continuous activity in a large population of single cells. Without intruding cell membrane using our approach, the cells could rely on intrinsic membrane potential-controlling mechanisms. The average calcium influx of these cells reached micromolar concentrations, demonstrating that our approach could evaluate low channel activity precisely. We observed that the Y511A mutant channel is still vanilloid-sensitive, but its response to capsaicin is weaker than that of wild-type. Therefore, we sought other mutations in residues near to Y511 that could disrupt vanilloid gating but preserve activation by non-vanilloid agonists, resulting in selection of the previously identified S512F mutation (Jordt and Julius, 2002). Although it behaves comparably to the wild-type TRPV1 receptor when challenged with 2-APB, the S512F mutant displays drastically lower capsaicin affinity. By assessing mutant channels, we determined that at least two of the four TRPV1 channel subunits has to be ligand-bound to evoke full agonist activation upon capsaicin or AEA administration. Our calcium imaging of HEK293T cell expressing wild type TRPV1 showed that capsaicin-induced calcium influx was at least two-fold greater than for AEA. This outcome is consistent with the previous finding that maximal AEA-induced current conductance was much lower than for capsaicin, based on whole-cell recordings of TRPV1 transiently-transfected HEK293 cells (Fenwick et al., 2017).

Binding site occupancy commonly drives channel opening to allow ion influxes *via* ligand-gated ion channels (Yang et al., 2015; Elokely et al., 2016). A tetrameric TRPV1 channel with two capsaicin-bound subunits is already sufficiently activated to raise cytosolic calcium to levels comparable to those induced in wild-type tetrameric TRPV1 receptors in which all four capsaicin-binding sites are occupied. Thus, it seems that TRPV1 sensing has evolved considerable redundancy so that it rarely fails. Oxidative sensitization of the TRPV1 channel also necessitates two cysteine-containing subunits to form the disulfide bond required to enhance channel sensitivity (Wang and Chuang, 2011; Ogawa et al., 2016). The N-terminus domains that mediate oligomerization of ion channel subunits may influence channel gating (Bhave et al., 2003; Rosenbaum et al., 2004), and generate the variation in TRPV1 activation apparent for differentially arranged subunit configurations. By covalently linking subunits, we could create all possible tetrameric arrangements comprising exactly one single wild type repeat, and all of those mutant channels could at least be partially opened by capsaicin. The requirement for binding of more than two ligands underlies a fundamental principle of TRPV1-related pain sensation. We emphasize that binding of the first ligand does not induce sufficient ion flux to generate a maximum TRPV1 response, thereby presumably safeguarding against overreaction in cases of inadvertent ligand encounters. We measured ligand-induced Ca^2+^ increases to represent stimulus strength-dependent activation. Single capsaicin molecule occupancy on a TRPV1 channel yields less than 10% full activation, thus eliciting weak stimulation unless the activated receptors on a cell were elevated to an unrealistically high number. The next level of response we observed for our dimers already represents a maximal TRPV1 response, with each assembled dimer-dimer channel binding two capsaicin molecules.

TRPV1 activation participates in both sensing and responding to pain. Receptor activation does not necessarily result in pain, instead operating over a wide dynamic range that includes sensations below the level of conscious awareness (Bhave et al., 2003), demonstrating remarkable economy in conveying both painful and innocuous sensations. The TRPV1 channel functions as a natural device for integrating painful stimulations, with graded responses reflecting stimulus intensity (Hui et al., 2003; Chuang and Lin, 2009; Gouin et al., 2017). Using an ionotropic receptor to signal pain ensures remarkable response speed and direct additivity. Processing the primary input signal *via* ion channels facilitates a simple and effective summation mechanism, which could be efficiently and speedily translated to alert the host of potential or actual physical harm. Our study highlights the potential for developing an objective metric to score pain modifiers that act through TRPV1, representing a crucial intermediate step towards efficacious and titratable pain medicine.

## Data Availability Statement

The datasets generated for this study are available on request to the corresponding author.

## Author Contributions

T-YL, YC, H-RM, and DC performed the calcium imaging. T-YL performed the Western blot. H-RM performed electrophysiological experiments. T-YL, YC, and H-RM designed the experiments, wrote and revised the manuscript. H-HC designed the experiments, supervised their progression, wrote and revised the manuscript, and communicated with editors.

## Conflict of Interest

The authors declare that the research was conducted in the absence of any commercial or financial relationships that could be construed as a potential conflict of interest.
